# FAX1 not to the max: Chloroplast export control on fatty acids during cold acclimation

**DOI:** 10.1093/plcell/koae046

**Published:** 2024-02-10

**Authors:** Renuka Kolli

**Affiliations:** Assistant Features Editor, The Plant Cell, American Society of Plant Biologists; Sainsbury Laboratory, University of Cambridge, Cambridge, UK

Membranes of plant cells contain 3 main types of lipids, namely glycerolipids, sphingolipids, and sterols. Glycerolipids are the most abundant and are especially crucial for the structure and function of photosynthetic complexes in the thylakoid membrane. Their structure comprises a glycerol backbone to which 2 varied fatty acid chains and a varied head group are typically attached. Fatty acids synthesized in a plastid proceed to glycerolipid synthesis via either the prokaryotic pathway within the plastid or the eukaryotic pathway in the endoplasmic reticulum (see Fig.). Different plant species have varied relative contributions from the 2 pathways. In 16:3 plants such as Arabidopsis, spinach, and tomato, about one-half of the chloroplast glycerolipids are derived from each of the pathways (Browse and Somerville 1991). On the other hand, in 18:3 plants such as wheat, maize, and pea, chloroplast glycerolipids are predominantly obtained from the eukaryotic pathway.

**Figure.**
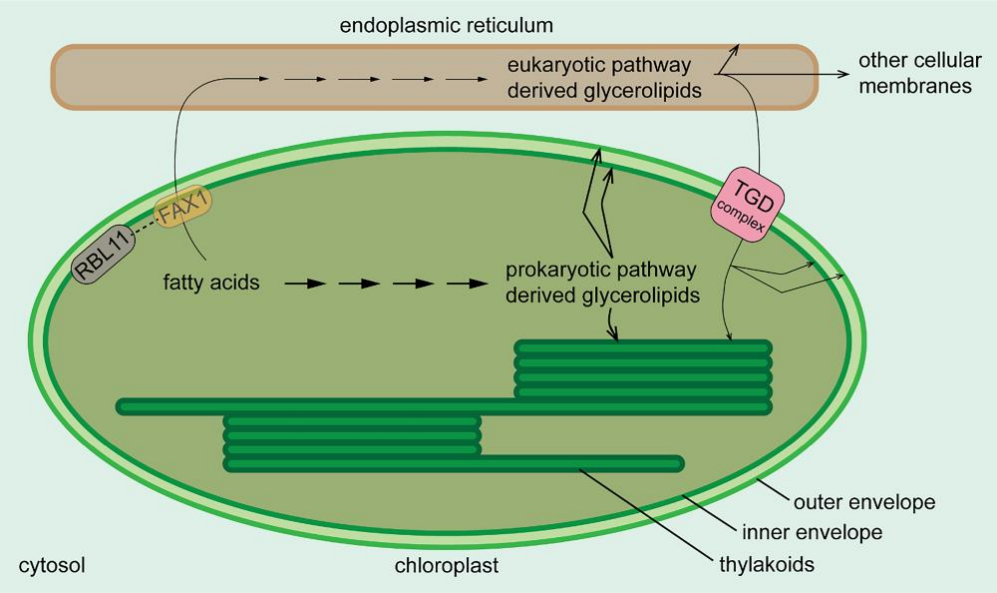
Schematic diagram of the 2 pathways of glycerolipid synthesis. RBL11-dependent FAX1 degradation reduces flux through the eukaryotic pathway (thinner arrows) in Arabidopsis during cold acclimation. Sequential arrows denote multiple reactions to produce a variety of glycerolipids. The arrows pointing to membranes denote the incorporation of the specified glycerolipids directly or upon further modification(s) into the indicated membranes. Figure credit: R. Kolli.

Low-temperature stress decreases membrane fluidity and enzyme activities that disrupt photosynthesis, thereby restricting plant growth and development (Liu et al. 2018). Many temperate plants including Arabidopsis develop freezing tolerance upon exposure to low nonfreezing temperatures for a few days. During the cold acclimation process, remodeling of membrane lipids mainly involves increasing the degree of fatty acid unsaturation and altering lipid composition to maintain membrane fluidity at low temperatures. Membrane remodeling is supported by adjusting the relative contributions of the 2 glycerolipid synthesis pathways. Moreover, when energy is limiting, the prokaryotic pathway is advantageous for chloroplast membrane remodeling, because it saves the energy required for fatty acid export and lipid import. Accordingly, in Arabidopsis, the prokaryotic pathway was promoted at low temperatures, whereas the eukaryotic pathway was promoted at high temperatures (Li et al. 2015b).

The Arabidopsis fatty acid exporter 1 (FAX1), localized in the inner envelope, is involved in exporting fatty acids from the chloroplast (Li et al. 2015a; see Fig.). FAX1 thus controls the input to the eukaryotic pathway. Numerous studies have shown that recombinant overexpression of FAX1 alone or in combination with other fatty acid transporters leads to enhanced oil production in plants and algae. Recently, Trentmann et al. (2020) found that FAX1 decreased in abundance when Arabidopsis plants were subjected to chilling. To investigate how FAX1 abundance decreases during chilling and whether this phenomenon contributes to plant cold tolerance, **Annalisa John and coauthors** (John et al. 2024) screened the mutants of inner envelope–localized proteases and performed extensive studies on FAX1 overexpression mutants. Several lines of evidence indicated that the rhomboid-like (RBL) protease RBL11 is likely involved in FAX1 degradation during chilling. Based on bimolecular fluorescence complementation, FAX1 interacted with RBL11 but not with FTSH11, which is another inner envelope protease. There was a lack of cold-induced FAX1 degradation in *rbl11* loss-of-function mutants. Tandem mass spectrometry analysis of glycerolipids showed that the *rbl11* loss-of-function mutants with increased FAX1 abundance and the FAX1 overexpression mutants exhibited a shift to the eukaryotic pathway of glycerolipid synthesis at low temperatures. Both the mutants were also impaired in cold and freezing tolerance compared with the wild-type plants, based on various parameters, including increased number of wilted leaves per plant, increased levels of reactive oxygen species and anthocyanins, and decreased plant survival rate. Therefore, the authors propose that RBL11-dependent FAX1 degradation during cold acclimation reduces flux through the eukaryotic pathway to support cold tolerance mechanisms in Arabidopsis (Fig.).

The trigalactosyldiacylglycerol (TGD) complex, spanning the outer and inner envelopes, imports eukaryotic pathway-derived glycerolipids into the chloroplast (Li et al. 2016; see Fig.). Interestingly, besides FAX1, 2 TGD complex subunits, TGD2 and TGD3, decreased in abundance upon chilling (Trentmann et al. 2020). Hence the decreased levels of FAX1, TGD2, and TGD3 might together reduce the eukaryotic pathway flux and favor the prokaryotic pathway for chloroplast membrane remodeling during cold acclimation. Future investigations could confirm this and study the eukaryotic pathway flux changes occurring in response to other stress conditions, such as drought and salinity.

## Dive Curated Terms

The following phenotypic, genotypic, and functional terms are of significance to the work described in this paper:

TGD3 Gramene: At1g65410
TGD3 Araport: At1g65410

fatty acid CHEBI: CHEBI:35366

FAX1 Gramene: At3g57280
FAX1 Araport: At3g57280

TGD2 Gramene: AT3G20320
TGD2 Araport: AT3G20320

FTSH11 Gramene: AT5G53170
FTSH11 Araport: AT5G53170

RBL11 Gramene: AT5G25752
RBL11 Araport: AT5G25752
